# Manipulating plant geometry to improve microclimate, grain yield, and harvest index in grain sorghum

**DOI:** 10.1371/journal.pone.0173511

**Published:** 2017-03-06

**Authors:** Sushil Thapa, Bob A. Stewart, Qingwu Xue, Yuanquan Chen

**Affiliations:** 1 Dryland Agriculture Institute, West Texas A&M University, Canyon, TX, United States of America; 2 Texas A&M AgriLife Research and Extension Center, Amarillo, TX, United States of America; 3 College of Agronomy, China Agricultural University, Beijing, China; Estacion Experimental del Zaidin, SPAIN

## Abstract

Cultivar selection, planting geometry, and plant population are the key factors determining grain sorghum yields in water deficit areas. The objective of this study was to investigate whether clump geometry (three plants clustered) improves microclimate within crop canopy when plants are grown under varying water levels. In a 2-yr sorghum (*Sorghum bicolor* L. Moench) greenhouse study, plants were grown at two geometries (clump and conventional evenly spaced planting, ESP), two water levels (high and low, representing well-watered and water-limited condition, respectively), and three soil surface treatments (lid covered, straw-mulched, and bare). Air temperature and relative humidity (RH) within the plant canopy were measured every five minutes at different growth stages. Mean vapor pressure deficits (VPDs) within the clumps were consistently lower than those for ESPs, indicating that clumps improved the microclimate. Clumps had significantly higher harvest index (HI) compared to ESPs (0.48 vs. 0.43), which was largely due to clumps having an average of 0.4 tillers per plant compared to 1.2 tillers per plant for ESPs. Grain yield in the current study was similar between clumps and ESPs. However, our results suggest that improved microclimate was likely a reason for clumps producing significantly higher grain yields compared to ESPs in previous studies.

## Introduction

Water scarcity and drought are the major constraints for crop production in many parts of the world [1, 2, 3]. Projections indicate that water availability for crops in some regions may be decreased due to increased patterns of erratic rainfall, lengthened intervals between rain events, and less rainfall during the crop growing season [4]. In such climates, grain sorghum (*Sorghum bicolor* L. Moench) is a desirable crop for growers because it is a drought-tolerant [5] and water-use-efficient cereal crop grown in semi-arid tropical and subtropical environments [6, 7, 8]. Despite its ability to tolerate drought, water deficit (WD) stress during booting and flowering stages results up to 85% reductions in grain yield [9].

The Texas High Plains is characterized by limited precipitation and high evaporative demand due to high wind speed, solar radiation, temperature, and vapor pressure deficit (VPD) [10, 11]. Because of the high VPD, the difference between the amount of moisture present in the air and how much moisture the air can hold when it is saturated [12], plants growing in the dry areas lose substantial amounts of water through transpiration. Increase in transpiration causes a decrease in the leaf surface temperature, but as WD stress occurs, plants close their stomata to avoid further water losses. As a result of decreasing transpiration cooling, leaf temperature increases [13]. Change in temperature influences the VPD and hence, microclimate within crop canopy. The importance of microclimate in determining crop performance is well established [14].

Manipulation of plant geometry such as reduced plant populations, wider plant or row spacing, and skip-row configurations are some of the strategies that have been adopted in dryland farming areas for better utilization of available soil-water [15]. However, decreased plant population might reduce water use efficiency (WUE) by exposing more leaf area per plant as well as soil surface to the environment. Growing three to four plants in clumps is a strategy based on the rationale that it will increase competition among the plants resulting efficient utilization of available soil water. Further, the vegetative mass will be reduced mainly because of less tillering [16]. Some previous studies suggested that, compared to conventional evenly spaced plating (ESP), growing grain sorghum and maize (*Zea mays* L.) in clumps increased the grain yield and harvest index (HI), mainly by reducing vegetative growth during early growth stages that conserved some soil water for reproductive and grain filling stages [11, 17, 18, 19]. Clump geometry creates a dense canopy and modifies the plant canopy architecture, the organization of plant components including its shape and size in space [20]. The canopy architecture influences the microclimate within crop canopy [21], which might be true in case of clump geometry as well, but it is not studied.

The hypothesis of this study was that growing sorghum plants in clumps would improve microclimate by mutual shading and exposing less leaf area per plant to the environment thereby decreasing the VPD within crop canopy. Our objective was to compare microclimate (VPD within plant canopy), grain yield, and yield components between clump (three plants clustered) and conventional ESP geometries at different water and soil surface treatments.

## Materials and methods

Experiments were conducted in the greenhouse of West Texas A&M University, Canyon, Texas in the summers of 2013 and 2014. Wooden boxes were used to grow grain sorghum (cultivar: DK-S36-06) in two geometries (clump and ESP), two water levels (high and low) and three soil surface types (lid covered, straw-mulched, and bare surface). For both years, night minimum and day maximum temperatures were maintained 20°C and 32°C, respectively. The RH for the growing period ranged from 28 to 95%. The light intensity of the greenhouse was about 1000 μmoles m^-2^s^-1^ PAR at noon. Light intensity, temperature, and humidity inside the greenhouse were verified similar at different locations. There were no other studies in the greenhouse at the same times.

### Experimental design

**Fig 1:**
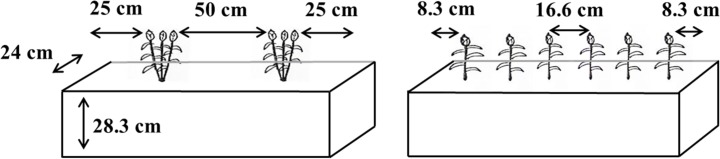
Arrangement of clump (left) and evenly spaced planting (ESP; right) geometries in the wooden boxes.

Thirty-six wooden boxes having a volume of 68 L (Fig 1) and filled with 46.3 kg of Calcined clay were used to grow sorghum. This material is porous, has a low bulk density (0.68 g cm^-3^ after packing), retains a large quantity of plant available water, is chemically inert and maintains good aeration and drainage properties needed for plant growth [22]. All boxes were brought to 42% volumetric water content by adding 28.6 L of filtered water. Before adding water, 75 g of “Miracle-Gro water soluble all-purpose plant food” was mixed uniformly in each box. This fertilizer provided N-P-K of 18, 2.6, and 10.0 g respectively, and some amounts of boron (B), copper (Cu), iron (Fe), manganese (Mn), molybdenum (Mo), and zinc (Zn). Potential water leakage was prevented by lining each box with a plastic sheet. Boxes were randomized 25 cm apart in a nested split plot design with three replications.

Sorghum seeds were planted on 15 May 2013 and 23 May 2014. Before planting, all the boxes were weighed using a common balance. In clump geometry, six plants were grown in two clumps (three plants in each clump) per box, which were 50 cm apart and 25 cm away from each end of the box. In ESP geometry, six plants were individually spaced 16.6 cm apart in a row, leaving 8.3 cm at either end of the boxes (Fig 1). Five to six seeds in clump and three to four seeds in ESP geometry were planted. When the seedlings reached 10–12 cm, they were thinned to three plants in each clump and individual plants in ESP.

In 2013, water was added to the boxes based on visual WD stress observed in plants at low water treatments. Before adding water, experimental boxes were weighed to monitor the amount of water used and determine how much should be added. At each watering, plants at low water treatment were provided with 50% less water than plants growing at the high water treatment. For all treatments, water was added on the same day. Precautions were taken not to exceed the volumetric water content of 42% for the high water treatment. This protocol caused plants at some low water treatment boxes (especially with bare soil surface) became stressed before plants at high water treatments (especially with lid covered surface) were in need of added water. Therefore, in 2014, volumetric water contents between 35–42% and 28–35% were maintained for high and low water treatments, respectively. To monitor the water use, every box was provided with a digital balance. Using this protocol, overall amounts of added water were higher than for the previous method, and plants at low water treatment received 26% less water than plants at high water treatment. A total of 46.4 L at high and 23.2 L at low water treatments were added in 2013 and in 2014, 61 L and 45 L were added for high and low water treatments, respectively.

For lid surface treatment, boxes were covered with wooden lids with holes for growing plants. The holes made for plants had an area of 7.9 cm^2^ for ESPs and of 20.3 cm^2^ for clumps. In order to prevent evaporation from the holes, they were covered with plastic tape, leaving a small portion sufficient for emerging seedlings. The tape was readjusted as the plants grew. All water lost from these boxes was assumed to be transpiration. The mulched treatment was covered with wheat straw of 4 Mg ha^-1^ [23].

### Measurements

Air temperature and relative humidity (RH) within the plant canopy were measured using the LASCAR EL-USB-2+ sensors at different growth stages, but the emphasis was given for the booting through gain formation period (45–70 DAP), which is considered most critical growth stages for WD stress in grain sorghum [9]. These sensors measure and store up to 16,382 RH (0 to 100%) and 16,382 temperature (-35 to +80°C) readings [24]. Each sensor was positioned vertically at the upper portion of the canopy height, where typically highest and presumably the greatest proportion of transpiration occurs [25]. Sensors were located close to the main stem without touching plant parts. The measurements were recorded from three replications, every five minutes continuously for three days in 2013 and five days in 2014. VPD was calculated using the following equations described by CronkLab [26]:

$$SVP=610.7\times 10^{7.5T/(237.3+T)}$$ (1)

$$\text{and}\;VPD=\left[\frac{100-RH}{100}\right]\times SVP$$ (2)

where SVP is saturated vapor pressure (kPa), T is temperature (°C), VPD is vapor pressure deficit (kPa), and RH is relative humidity (%).

All the plants were included in counting tiller number at flag leaf stage, and then leaf area per plant (including tillers) was measured using:

LA=W×L×0.75 (3)

where LA is the leaf area (cm^2^), W is the maximum width (cm) of the leaf, L is the leaf length (cm) from leaf collar to the end of the leaf tip, and 0.75 is a correction factor (*k*) for sorghum [27, 28].

The sorghum plants were harvested on 08 Sept. (118 days after planting, DAP) in 2013 and on 16 Sept. (116 DAP) in 2014. Before harvesting, all the boxes were weighed on the common balance in order to calculate the evapotranspiration (ET) as:

$$V=(w_{i}+v_{t})-w_{f}$$ (4)

where V is the cumulative volume (L) of water used in ET, w_i_ is the initial weight (kg) of a box at seeding, v_t_ is the total volume (L) of water added during the crop growing period, and w_f_ is the final weight (kg) of a box before crop harvest. Since water has density of 1 g cm^-3^ (i.e. 1 g ml^-1^), weight of water is equivalent to its volume.

Plant samples were dried in the oven at 70°C until the constant weight was recorded. After drying, they were weighed to obtain the aboveground biomass and threshed to measure grain yields. The biomass transpiration efficiency (TEb) and grain transpiration efficiency (TEg) for the lid surface treatment was calculated by dividing the weight of total aboveground dry biomass and grain yield, respectively, by the total amount of transpiration. Similarly, biomass water use efficiency (WUEb) and grain water use efficiency (WUEg) were calculated by dividing aboveground dry biomass and grain yield, respectively, by total amount of water used in ET.

## Statistical analysis

Data were analyzed via two-way analysis of variance (ANOVA) using PROC MIXED in SAS 9.3 [29]. Year, planting geometry, water level, and surface type were considered as fixed effects. Replication was a random effect. The mean separation test was done using least significance difference (LSD), and differences were considered significant at *P* < 0.05. The PROC REG in SAS 9.3 was used to develop regression coefficients between aboveground biomass or grain yield and water transpired from the plants in lid surface treatment.

## Results and discussion

### 2013 and 2014 experiments

Leaf area, aboveground biomass, and grain yield were significantly (*P* < 0.05) higher in 2014 than in 2013 (Tables 1 and 2). The difference increased from high water to low water, and lid to straw mulch to bare surface treatments (Table 3). It was mainly because watering was done adopting different methods in 2013 and 2014. Compared to 2014, in 2013 total water added was lower by 14.6 L (23.9%) at high water and 21.8 L (48.4%) at low water treatments. Lower WUE in 2013 might be associated with the higher evaporative loss, especially from straw mulched and bare surface treatments, due to frequent and small amount of irrigation. However, HI remained similar for both years (Tables 1 and 2).

**Table 1:** P-values of sorghum leaf area, tiller number, aboveground biomass (AGB), grain yield, harvest index (HI), biomass water use efficiency (WUEb), and grain water use efficiency (WUEg) as affected by year, geometry, water, and soil surface as determined by analysis of variance (ANOVA).

Effect	Leaf area	Tiller number	AGB	Grain yield	HI	WUEb	WUEg
Year (Y)	< .0001	-§	< .0001	< .0001	0.1602	< .0001	< .0001
Geometry (G)	< .0001	< .0001	0.0045	0.2435	0.001	0.0273	0.4073
Water (W)	.0011	0.6807	< .0001	< .0001	0.0016	0.0003	<0.0001
Surface (S)	< .0001	0.0021	< .0001	< .0001	0.7844	< .0001	< .0001
Y×G	< .0001	-	0.1029	0.4912	0.2235	0.4108	0.7790
Y×W	0.0011	-	0.0013	0.0034	0.0505	0.0362	0.6005
Y×S	< .0001	-	0.3332	0.1824	0.0439	< .0001	0.7958
G×W	0.4571	0.5948	0.4096	0.9710	0.6828	0.7752	0.8363
G×S	0.3107	0.2099	0.9924	0.3877	0.8688	0.9670	0.7958
W×S	0.5305	0.4412	0.0134	0.0013	0.8997	0.0023	0.0153
Y×G×W	0.4571	-	0.9831	0.7854	0.9042	0.9191	0.9789
Y×G×S	0.3107	-	0.8562	0.7128	0.9638	0.7556	0.9465
Y×W×S	0.5305	-	0.0277	0.0005	0.1510	0.0023	< .0001
G×W×S	0.3370	0.6068	0.9968	0.6115	0.5431	0.9752	0.8007
Y×G×W×S	0.3370	-	0.6996	0.9467	0.5998	0.7910	0.8667

§Tiller data was not obtained for 2013 due to very fewer tillers.

**Table 2:** Means of Leaf area, tiller number, aboveground biomass (AGB), grain yield, harvest index (HI), biomass water use efficiency (WUEb), and grain water use efficiency (WUEg) of sorghum grown in 2013 and 2014 at two planting geometries, two water levels, and three soil surface types.

Effect	Leaf area per plant (cm^2^)	Tiller number (plant^-1^)	AGB (g box^-1^)	Grain yield (g box^-1^)	Harvest index‡	WUEb (kg m^-3^)	WUEg (kg m^-3^)
Year							
2013	778.9 b†	-	160.8 b	72.4 b	0.44 a	3.34 b	1.48 b
2014	1430.8 a	-	273.4 a	125.1 a	0.46 a	3.99 a	1.83 a
Geometry							
Clump	988.0 b	0.4 b	210.4 b	100.3 a	0.48 a	3.57 b	1.67 a
ESP	1221.8 a	1.2 a	223.8 a	97.2 a	0.43 b	3.77 a	1.64 a
Water							
High	1248.4 a	0.9 a	261.8 a	122.4 a	0.47 a	3.87 a	1.89 a
Low	961.4 b	0.8 a	172.5 b	75.1 b	0.44 b	3.47 b	1.42 b
Surface							
Lid	1532.3 a	1.0 a	301.6 a	136.5 a	0.45 a	5.33 a	2.36 a
Straw	1127.4 b	1.1 a	218.0 b	98.1 b	0.45 a	3.60 b	1.64 b
Bare	654.9 c	0.4 b	131.8 c	61.7 c	0.46 a	2.08 c	0.97 c

N = 36, means are the average of three replications.

†Within each effect and each column, means with the different letter are significantly different at P < 0.05.

‡Harvest index is based on dry weight of grain divided by dry weight of aboveground biomass.

**Table 3:** Means of aboveground biomass (AGB), grain yield, biomass water use efficiency (WUEb), and grain water use efficiency (WUEg) of sorghum grown in 2013 and 2014 at two water levels and three surface types.

Year	Water level	Soil surface	AGB (g box^-1^)	Grain yield (g box^-1^)	WUEb (kg m^-3^)	WUEg (kg m^-3^)
**2013**	High	Lid	303.4 a†	135.8 a	5.26 a	2.35 a
** **		Straw	224.1 b	112.0 b	3.76 b	1.88 b
** **		Bare	112.1 c	52.5 c	1.90 c	0.89 c
** **		**Mean**	**213.2 B**‡	**100.1 B**	**3.64 AB**	**1.71 AB**
** **	Low	Lid	195.7 a	80.8 a	5.55 a	2.30 a
** **		Straw	91.5 b	38.3 b	2.56 b	1.07 b
** **		Bare	38.1 c	15.2 c	1.05 c	0.42 c
** **		**Mean**	**108.4 C**	**44.8 C**	**3.05 B**	**1.26 B**
**2014**	High	Lid	398.2 a	194.9 a	5.35 a	3.06 a
		Straw	311.7 b	134.9 b	4.09 b	1.82 b
		Bare	221.3 c	104.5 c	2.86c	1.37 c
		**Mean**	**310.4 A**	**144.8 A**	**4.10 A**	**2.08 A**
	Low	Lid	309.0 a	134.6 a	5.17 a	1.74 a
		Straw	244.7 b	107.1 b	3.98 b	1.79 a
		Bare	155.6 c	74.5 c	2.50 c	1.21 b
		**Mean**	**236.4 B**	**105.4 B**	**3.88 AB**	**1.58 B**

N = 36, means are the average of three replications.

†Within each water level in each column for each year, means with the different lowercase letter are significantly different at P < 0.05.

‡In each column, means with the different uppercase letters are significantly different at P < 0.05.

### Vapor pressure deficit

**Fig 2:**
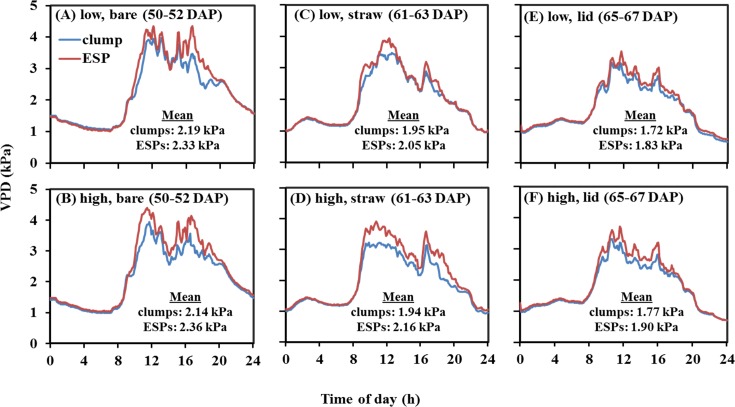
Average 3-day vapor pressure deficit (VPD) mean within the plant canopy recorded every five minutes for different treatments in 2013 at 50–52, 61–63, and 65–67 DAP corresponding to booting, flowering, and grain formation growth stages, respectively. DAP: days after planting; ESPs: evenly spaced plantings. The mean VPD is derived from the average of 2592 data points.

**Fig 3:**
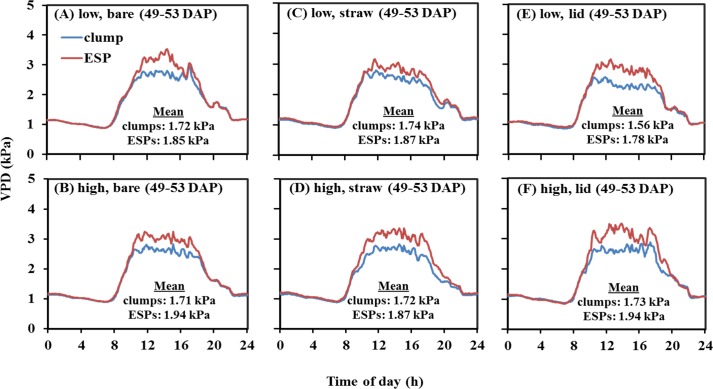
Average 5-day vapor pressure deficit (VPD) mean within the plant canopy recorded every five minutes for different treatments in 2014 at 45–53 DAP corresponding to booting growth stage. DAP: days after planting; ESPs: evenly spaced plantings. The mean VPD is derived from the average of 4320 data points.

For both years, plants grown under clump geometry consistently showed lower VPD within the canopy as compared to those under ESP geometry, though the VPD values and differences varied with time of day (Figs 2 and 3). VPDs for clumps and ESPs did not differ during the night hours, but as the day progressed, different VPDs were observed. In most cases, the maximum VPD was found about 11:00 a.m. central standard time (CST), though it was not the hottest part of the day. This was because as the temperature rose (close to 32°C), the greenhouse shade closed and cooling fan started to circulate cool and moist air which decreased temperature and increased humidity. This process occurred continuously throughout the day, resulting in the fluctuation of temperature and humidity, hence the VPD. For 2013, VPD for different soil surface treatments was recorded at different growth stages (i.e. booting, flowering, and grain formation; Fig 2) due to the lack of sufficient number of sensors, but for 2014, VPD for all surface types was measured at the same growth stage (i.e. booting; Fig 3).

In 2013, with bare soil surface, at low water, clump showed the mean VPD of 2.19 (±0.05 se) kPa and ESP showed 2.33 (±0.06 se) kPa, and at high water, clump had the mean VPD of 2.14 (±0.5 se) kPa which was lower than 2.36 (±0.05 se) kPa in ESP (50–52 DAP; Fig 2A and 2B). Similar trends of lower mean VPDs for clumps compared to those for ESPs were recorded with straw mulch surface (61–63 DAP; Fig 2C and 2D) and lid surface (65–67 DAP; Fig 2E and 2F) both at low and high water treatment. In 2014, with bare soil surface, clump and ESP had the mean VPD of 1.72 (±0.04 se) kPa and 1.85 (±0.05 se) kPa, respectively at low water, and at high water, clump had the mean VPD of 1.71 (±0.04 se) kPa and ESP had 1.94 (±0.05 se) kPa (49–53 DAP; Fig 3A and 3B). As in case of bare soil surface, similar trends of lower mean VPDs for clumps than those for ESPs were found with straw mulch surface (49–53 DAP; Fig 3C and 3D) and lid surface (49–53 DAP; Fig 3E and 3F) both at low and high water treatment. Since the measurements were taken during the same DAPs in 2014, they can be compared from one surface type to the other. Overall, ESPs had VPDs greater by 5–14% than clumps.

At water stressed environment, the stomata close and increase the leaf temperatures [30, 31, 32, 33]. Since closing stomata disrupts photosynthesis, under water-limiting conditions, plants having less leaf area with opened stomata are better than plants having more leaf area with closed stomata on some or all of the leaves [34, 35]. In this study, clump geometry reduced leaf area per plant significantly (*P* < 0.05; 1221.8 vs. 988.0 cm^2^ for ESPs), which probably was useful in keeping stomata opened thereby reducing the temperature and increasing the humidity within crop canopy. Visual WD stress symptoms were more apparent for the plants in ESPs compared to the plants in clumps, because increased leaf area also increased transpiration and decrease soil moisture faster, which was also reported by Rajan et al. [35]. Further, losing more water through transpiration in ESPs might trigger stomata to be closed and increased VPD within the crop canopy. Because shade improves the microclimate by keeping plants cooler during the day and warmer at night [36], sorghum plants in clump geometry might be benefited from mutual shading, which helped to reduce the VPD. In our maize (*Zea mays* L.) field study too, mean VPDs within crop canopy were lower for clumps than those for ESPs, when measured at reproductive and grain filling growth stages [11].

### Number of tillers

Tiller data were not obtained for 2013 because few were formed, while in 2014, ESPs produced significantly (*P* < 0.05) more tillers (1.2 tillers plant^-1^) compared to the clumps (0.4 tillers plant^-1^; Tables 1 and 2). Out of the productive tillers (which produced harvestable grains), per tiller grain yield was greater for clumps (3.2 g tiller^-1^) than those for ESPs (2.1 g tiller^-1^). The mean tiller number was not different (*P* > 0.05) between high (0.9 tillers plant^-1^) and low (0.8 tillers plant^-1^) water treatments because both were started with 42% volumetric water content and most of the tillering occurred at the early vegetative growth stage when soil-water was not a limiting factor. Since low water treatment was provided with 26% less water than high water treatment, the percentage of tillers that produced harvestable grains was significantly higher at high water, 66.7% of total tillers compared to 28.7% at low water treatment. For surface types, plants with lid (1.0 tiller pant^-1^) and straw (1.1 tillers plant^-1^) surface had a similar number of tillers, while plants with bare surface had significantly (*P* < 0.05) fewer (0.4 tillers plant^-1^; Table 2).

Tillers are formed because of the activity of the axillary meristem in the axils of the leaves adjacent to the main stem [37, 38, 39]. Lafarage and Hammer [40] observed that tiller emergence was driven by tiller site formation at the base of every leaf, and by the number of buds that develop into tillers. Where water and nitrogen are not limiting factors, tiller production is affected by plant carbon balance and in particular the availability of assimilates [41]. A low light interception, a short photoperiod, or high planting density reduces the assimilate supply [37, 42]. Less available growing space in a dense canopy also decreases tillering [43, 44]. These were likely to be the reasons for having lesser number of tillers in clumps compared to ESPs in the present study. Tiller emergence is also related to the light quality [45]. For instance, the production of tillers or branches reduced as the red light to far-red light ratio (R:FR) is decreased [42, 46, 47, 48, 49]. Because three plants were grown together in each clump, they might allow lower R:FR light ratio reaching at the base of the plants resulting less tiller formation.

### Biomass, grain yield, and harvest index

Aboveground biomass was significantly (*P* < 0.05) affected by all main effects and year × water, water × surface, and year × water × surface interactions (Table 1). The mean biomass amounts for clumps and ESPs were 210.4 g box^-1^ and 223.8 g box^-1^, respectively, indicating that ESPs produced 13.4 g box^-1^ (6.4%) more biomass compared to clumps (Table 2). The soil surface showed different biomass production in response to water levels. Average biomass production across high and low water treatments in 2013 and 2014 were lid surface (301.6 g box^-1^) > straw surface (218.0 g box^-1^) > bare soil surface (131.8 g box^-1^; Table 2), and the difference was larger at low water compared to high water treatment (Table 3). The grain yield was significantly (*P* < 0.05) affected by the year × water × surface, year × water, and water × surface interactions and all main effects except geometry (*P* > 0.05; Table 1). For both years and water treatments, plants growing with lid surface produced higher grain yields followed by plants with straw mulch and bare soil surface (Table 3). Although there was no statistical difference (*P* > 0.05), mean grain yield was relatively higher for clumps (100.3 g box^-1^) than for ESPs (97.2 g box^-1^; Table 2). The HI was significantly (*P* < 0.05) affected by the main effects of plating geometry and water (Table 1). Clumps had higher HI, 0.48 compared to ESPs, 0.43. Plants growing at high water had the HI of 0.47 which was higher than the HI of 0.44 for plants at low water treatment (Table 2).

Planting pattern can lead to changes in the microclimate environment, especially light interception, sunshine hours, temperature, and ET within crop canopy [50]. In addition, other natural properties such as LAI and final production are also affected [51]. Bandaru et al. [17] grew grain sorghum in Bushland, Texas and reported that aboveground biomass at harvest was significantly lower for the clumps compared to ESPs which was verified by our study. In the same location, however, Kapanigowda et al. [52] found a significantly higher aboveground biomass in clumps compared with ESPs at harvest in maize. This might be because clump geometry was found efficient in reducing the number of tillers, and the tiller production has been reported more common in sorghum than in corn. In both of these studies, clump geometry produced higher grain yields than that of ESP. Sorghum grain yield also increased when plants were grown in closer row spacing [53]. Though HI for sorghum has a genetic potential of about 0.53 [54], it decreases sharply with increasing WD stress [55]. In our study, improved microclimate within the crop canopy helped clumps to delay or lower the WD stress, which positively contributed to grain yield and HI. In contrast, increased tiller numbers coupled with WD stress at reproduction and grain filling periods might be the reason for decreasing grain yield relatively and HI significantly in ESPs compared to clumps. Previous studies also reported higher HI in clumps than in ESPs, when sorghum and maize were grown under semi-arid climatic environments [11, 18, 19, 37, 52]. Hence, results indicate that to take the full advantage of climatic conditions, the appropriate choice of planting geometry is essential.

### Transpiration efficiency and water use efficiency

**Fig 4:**
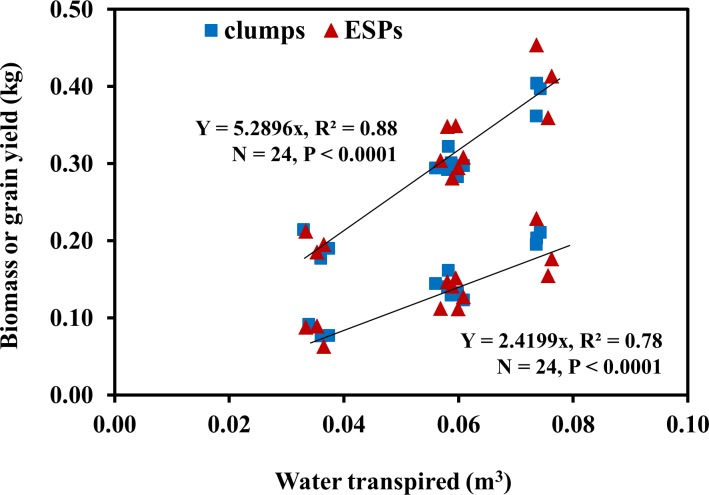
Linear regression between water transpired, and aboveground biomass or grain yield for plants grown with lid covered surface treatments in 2013 and 2014. TEb: biomass transpiration efficiency; TEg: grain transpiration efficiency (N = 24).

Neither TEb nor TEg was significantly (*P* > 0.05) affected by the main effects (year, geometry, and water) or their interaction; hence, a common regression line is used to represent both clumps and ESPs for each of TEb and TEg. On average, plants produced 5.29 kg and 2.42 kg of dry biomass and grain yield, respectively for each cubic meter of water transpired (Fig 4).

WUEb and WUEg were significantly (*P* < 0.05) affected by year × surface, water × surface, and year × water × surface interactions and all main effects except geometry (*P* > 0.05) for WUEg (Table 1). WUEb was significantly higher for ESPs (3.77 kg m^-3^) than for clumps (3.57 kg m^-3^), but WUEg was relatively higher for clumps (1.67 kg m^-3^) than for ESPs (1.64 kg m^-3^), and it was reflected as higher HI for clumps than for ESPs. High water treatment significantly (*P* < 0.05) increased both WUEb and WUEg compared to low water treatment. There was 42.2% reduction in WUEb and 40.7% reduction in WUEg when soil surface was changed from straw mulch to bare (Table 1).

Previous studies have shown a linear relationship between crop dry biomass and total water transpired by crops during the growing season [56, 57, 58, 59]. In our study too, as shown in Fig 4, the aboveground biomass (R^2^ = 0.88, *P* < 0.0001) and grain yield (R^2^ = 0.78, *P* < 0.0001) increased linearly with cumulative water transpired. Sorghum in the present study produced 1 g of biomass for each 189 g of water transpired (i.e. 5.29 kg m^-3^) in the mean VPD ranging from 1.56 to 2.36 kPa, which was close to the findings of Sinclair and Weiss [60]. They reported that C_4_ crops grown in an “average” transpiration environment of 2 kPa VPD will produce 1 g of biomass for every 220 g of water transpired, but for an arid region with a transpiration environment of 2.5 kPa VPD, crops use about 280 g for each g biomass. Because of the expected lesser VPDs within clumps, it was assumed that the TEb and TEg for clumps would be higher than for ESPs, but both of these values were similar between clumps and ESPs. It might be because the transpiration was measured only from the lid surface treatments, where plants in both clumps and ESPs were never water stressed. In both years and both water levels, compared to bare soil surface, straw mulch significantly increased WUEb and WUEg by reducing evaporative loss, which was reported in previous studies [23, 61, 62, 63].

## Conclusions

Vapor pressure deficit within crop canopy was consistently lower for clumps than that for ESPs in both years under different water levels and soil surface types. This was probably because of more mutual shading in clumps and hence, less leaf area exposure per plant to the environment. Since the number of tillers and vegetative mass were significantly reduced in clumps compared to ESPs, clumps were likely able to partition more of the biomass to grain formation increasing the HI. There was no overwhelming evidence to suggest that clump geometry would result in large changes in crop yields. However, compared to conventional ESP, clumps had improved microclimate, lesser vegetative mass, and higher HI, which are important attributes to be considered while growing crops in semi-arid climatic conditions. Hence, clump geometry appears to be a potential alternative for large scale implementation, which requires no additional input cost.
